# Roles of survival situation and personality temperament in the relationship between life stress and depression of higher vocational college students

**DOI:** 10.1186/s40359-023-01214-2

**Published:** 2023-05-30

**Authors:** Dong-Hui Cao, Lin-Ke Zheng

**Affiliations:** 1 School of Psychology, Shaanxi Normal University, NO. 199 Chang’an South Road, Yanta District, Xi’an, 710062 Shaanxi Province China; 2 Shaanxi Universities Psychological Quality Education Research Institute, NO. 563 Chang’an Road, Yanta District, Xi’an, 710061 Shaanxi Province China

**Keywords:** Higher vocational students, Life stress, Survival situation, Personality temperament, Depression

## Abstract

**Background:**

Higher vocational college students face more life stress, which can easily result in depression and hinder their healthy growth. This study aimed to explore the roles of survival situation and personality temperament in the relationship between life stress and depression.

**Methods:**

A self-compiled "College Students' Life Stress and Mental Health Questionnaire" was used to survey 4800 students in a Chinese higher vocational college. The questionnaire consisted of five subscales: life stressors scale, stress response scale, depression scale, personality temperament types scale, and survival situations scale. The sample included 4705 students, of whom 3449 (73.30%) were males and 1256 (26.70%) were females, with 990 urban students (21.04%), 3715 rural students (78.96%). The age of the participants ranged from 17 to 33 years. The data were analyzed using SPSS v26, PROCESS v3.3, and AMOS v23.

**Results:**

(1) The depression rate of higher vocational students was 18.10% (with a severe depression rate of 1.60%). Life stress could explain 43.80% of depressive episodes (*p* < 0.01), (2) Among survival situations, the depression degree and rate of students in adversity were the highest (M = 1.56, 24.10%), (3) Among temperament types, the depression degree and rate of melancholic students were the highest (M = 2.13, 36.05%), (4) Survival situation and personality temperament had significant moderating interaction effects on depression caused by life stress (*p* < 0.01), students in adversity and depressive temperament were more susceptible, (5) Survival situations moderated three paths of the "life stressors-stress response-depression" partial mediation model, and personality temperament types moderated "stress response-depression" path.

**Conclusion:**

Prosperity and sanguine temperament are protective factors of depression caused by life stress in higher vocational students. Dilemma, adversity and melancholic temperament are risk factors of depression caused by life stress in higher vocational students.

## Background

As is well-known, depression is an prevalent trend and has become a public health enemy [1–3]. Depression is a state of low mood and sense of meaninglessness. Its core symptoms are sense of worthlessness, helplessness, hopelessness and other emotional disorders, accompanied by a range of physical and psychological discomforts [4]. Relevant studies have shown that the depression rate among vocational college students is about 20.00% [5, 6]. Faced with this phenomenon, “how to relieve the depression of higher vocational college students” has become an urgent problem to be solved. At present, most of the research objects are undergraduate students, and less attention is paid to students in higher vocational colleges. In fact, higher vocational students account for half of the students in colleges and universities. With the increasing demand for technical talents in China, their mental health is particularly important. Taking higher vocational college students as the research object, this study tries to provide reference and thinking for relieving their depression.

Rosenthal [7] proposed the diathesis-stress theory of depression, which was later developed and expanded by Monroe and Simons [8]. Diathesis refers to a psychological quality, while stress includes two components: life stressors and stress response. According to the theory, the effects of diathesis and stress on depression can be classified into an additive model and an interactive model. The additive model suggests that diathesis and stress have cumulative effects on depression, and the combination of the two triggers depression. Depression can occur as long as one of them is extreme enough, even if the other is zero. The interactive model, on the other hand, suggests that the two factors are necessary for each other and interact to induce depression [9]. While The additive model is easy to explain, it is relatively simple and does not match reality well. In contrast, according to the view of the interaction of internal and external factors [10], the interactive model is more realistic, but requires more careful interpretation.This study was carried out based on the second model, with a aim of relieving depression among higher vocational college students from a more comprehensive and systematic perspective.

### Relationship between life stressors and depression

Life stressors refer to pressures that one faces in daily life. Existing studies have confirmed that life stressors have a significant direct effect on depression [11–13]. Higher vocational college students face a variety of life stressors, such as academic pressure [14], employment pressure [15], and social prejudice (e.g. “no future for vocational college students”) [16, 17], which can easily lead to psychological distress and depression.

### Mediating role of stress response

Stress response is an individual's adaptive reaction to life stressors. When facing stressors, people develop physical, emotional, or cognitive responses that allow them to effectively cope with stressors and achieve physical and mental balance. It is closely related to people's mental health levels (e.g. well-being, depression) [18]. Several studies have suggested that the mediating role of stress response between life stressors and depression.

### Moderating roles of survival situation and personality temperament

Survival situation refers to the environment in which an individual grows. One’s environment is always changing, such as entering higher vocational colleges, getting employed, getting married, having children, and experiencing epidemic. He or she will experience prosperity, dilemma, and adversity in his or her life. Several studies have shown that the risk of depression greatly increases in dilemma and adversity [18–20]. Current studies suggest that the moderating effect of survival situation. Personality temperament is a stable and consistent individual characteristic [21] and an important psychological diathesis [22] closely related to depression. Present studies have shown that different personality characteristics have a significant moderating effect on depression [23–26]. Current research has confirmed the moderating roles of survival situation and personality temperament, but the mechanism of them in the relationship between life stress and depression remains unclear and needs further exploration.

## Research hypothesis

This study aimed to explore the combined effects of personality temperament and survival situation in the process of depression caused by life stress, with the intention to bridge the gaps in literature and guide mental health education in higher vocational colleges. In this study, a scientific scale was used to measure life stress (including life stressors and stress response), personality temperament, survival situation and depression of students in a higher vocational college. Subsequently, a theoretical model among the five variables was constructed.

**Fig. 1:**
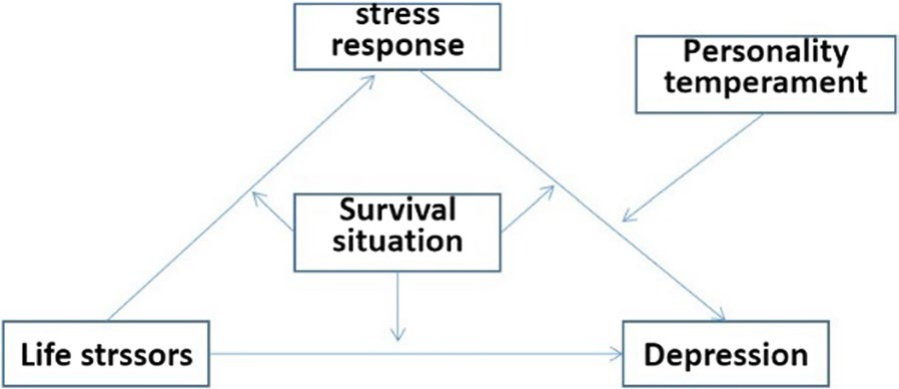
The proposed moderated mediation model

Based on the existing research, we formulate the following research hypothesis:

1. The depression rate among vocational college students is relatively high, estimated to be around 20.00%.
2. Students with different survival situations and personality temperament types exhibit varying rates and degrees of depression.
3. Survival situation and personality temperament play moderating roles in the "life stressors-stress response-depression" mediating model (see Fig. 1). Survival situation moderates the three paths of the mediating model. Personality temperament enhances or weakens depression by regulating stress response, and it moderates the "stress response-depression" path of the mediating model.

## Methods

### Participants

From April to May 2022, 4800 students from a higher vocational college in Shaanxi Province, China, were surveyed online. After eliminating extreme values, 4705 students were included in the study. The sample was derived from 6 secondary colleges and comprised 3449 boys (73.30%) and 1256 girls (26.70%), with 990 urban students (21.04%), 3715 rural students (78.96%), and an age distribution of 17–33 years old. Ethical approval was obtained from the ethics committee at the researcher's college.

### Procedure

To ensure the quality of the survey, 15 counselors from 6 secondary colleges underwent training before administering. They were taught about the meaning of the guiding words and matters needing attention during the training. Students clicked on an online link on a class basis. The testing process comprised four parts: explaining guiding words, collecting students' agreement, conducting the survey, and submitting the results. The data were then collated for further analysis.

### Instrument

A Self-compiled "College Students' Life Stress and Mental Health Questionnaire" was adopted, which was compiled in Chinese by Lin-ke Zheng, the second author of this study. This questionnaire includes five sub-scales of life stressors (LS), stress response (SR), depression (D), personality temperament types (PTT) and survival situations (SS).

The LS scale consists of 4 dimensions(Frustration stimulation, life changes, multiple loading, self-imposed) with 16 items, which is an equal interval scale. It has a total score, its cronbach’s alpha coefficient was 0.824, and KMO was 0.910 in the study, indicating that the reliability and validity of the scale were good. Each item has two answers (yes and no). The score for yes is 1, and the score for no is 0. The total score of each dimension represented the severity of the corresponding symptom, and the total score of the scale represented the amount of life pressure.

The SR scale consists of 4 dimensions (physiological response, emotional response, behavioral response, cognitive response) with 16 items, which is an equal interval scale. It has a total score, and its Cronbach’s alpha coefficient was 0.837 and KMO was 0.915 in the study, indicating that the reliability and validity of the scale were good. Each item has two answers (yes and no). The score for yes is 1, the score for no is 0. The total score of each dimension represented the severity of the corresponding symptom, and the total score of the scale represented the amount of stress response.

The D scale is an interval scale, which consists of multiple-choice questions with a total of 9 options (loss of interest, decreased energy, mental retardation, self-reproach, associative suffering, repeated death wishes, disturbed sleep, difficulty eating, decreased sexual interest). Each option is worth one point, and the total score is determined by the number of options selected. Its Cronbach’s alpha coefficient was 0.787 and KMO was 0.873 in the study, indicating that the reliability and validity of the scale were good.

The PTT scale is an ordinal scale with 2 dimensions (intro/extroversion, emotional in/stability), which grouped students into four temperament types: bilious temperament, sanguine temperament, phlegmatic temperament and melancholic temperament). Extroversion and emotional stability combine to become sanguine temperament, extroversion and emotional instability combine to become bilious temperament, introversion and emotional stability combine to become phlegmatic temperament, and introversion and emotional instability combine to become melancholic temperament.

The SS scale is an ordinal scale with nine levels, the higher the level, the worse the environment is perceived to be. Grades 1–3 are classified as prosperity, grades 4–6 as dilemma, and 7–9 as adversity.

### Data analysis

- **Step 1** According to the total score of depression, students was graded into 4 degrees and grouped into 2 groups, whose frequencies were calculated.
- **Step 2** The correlations between variables were analyzed.
- **Step 3** Analysis of variance and Chi-square test were used to judge whether there were significant differences in depression scores and rates among students with different personality temperament types and survival situations.
- **Step 4** A formula for the relationship between life stress and depression was established through binary logistic regression.
- **Step 5** Univariate analysis of General Linear Model was used to determine the moderating interactive effect of different temperament types and survival situations on depression caused by life stress.

Step 1–5 were all implemented using SPSSv26.

- **Step 6** AMOSv23 was used to verify the partial mediation model of "life stressor–stress response–depression" and the moderating effects of different personality temperament types and survival situations on the mediation model.
- **Step 7** Model 76 in PROCESSv3.3 was used to test the proposed model.

## Results

### Frequencies analysis

To test the first hypothesis, the present study conducted a frequencies analysis using SPSS-Analyze-Descriptive-Frequencies to examine the distribution of students across different levels and groups of depression. The severity of depression was ranked into four degrees from weak to strong: healthy level (y ≤ 2), mild depression (2 < y ≤ 5), medium depression (5 < y ≤ 7), Severe depression(y > 7). Based on their depression scores, students were dichotomized into two groups: the depression group (y > 2) and the non-depression group (y ≤ 2). As presented in Table 1, the results indicate that (1) 62 students (1.30%) were classified as severely depressed, 130 students (2.80%) medium depression, 661 students (14.00%) experienced mild depression, and 3852 students (81.90%) exhibited no signs of depression, (2) the number of students in the depression group was 853, which accounted for 18.10% of the total sample.

**Table 1:** Frequencies analysis of depressive degrees and groups

	Degrees/groups	Frequency	Percent	Cumulative percent
4 degrees of depression	Severe depression (y > 7)	62	1.30	1.30
	Medium depression (5 < y ≤ 7)	130	2.80	4.10
	Mild depression (2 < y ≤ 5)	661	14.00	10.60
	Healthy level (y ≤ 2)	3852	81.90	100.00
2 groups of depression	non-depression group (y ≤ 2)	3852	81.90	81.90
	depression group (y > 2)	853	18.10	100.00
	Total	4705	100.00	

y indicates total score of depression

### Correlations analysis of the variables

Previous research has suggested that life stressors, stress response, personality temperament, and survival situation are strongly associated with depression. To examine these relationships, the present study conducted a bivariate correlation analysis using SPSS-Analyze-Correlate–Bivariate correlation–Spearman. As shown in Table 2, the results indicate that (1) life stressors were positively correlated with depression, with a significant correlation coefficient (r = 0.566, *p* < 0.01), (2) life stressors were positively correlated with stress response (r = 662, *p* < 0.01), (3) stress response was significantly correlated with depression (r = 0.663, *p* < 0.01), (4) survival situation was significantly correlated with depression (r = 0.099, *p* < 0.01), (5) personality temperament was significantly correlated with depression (r = 0.166, *p* < 0.01).

**Table 2:** correlations analysis of the variables

	LS	SR	SS	PTT	D
LS	1.000				
SR	0.662**	1.000			
SS	0.145**	0.105**	1.000		
PTT	0.158**	0.160**	− 0.004	1.000	
D	0.566**	0.663**	0.099**	0.166**	1.000

*LS* life stressors, *SR* stress response, *D* depression, *SS* survival situations, *PTT* personality temperament types

**p < 0.01 (2-tails), indicating significance at the 1% level, the same below

### Analysis of variance and Chi-square

To investigate the effects of survival situation and personality temperament on depression, the present study conducted a one-way ANOVA using SPSS Analyze—Compare means. As displayed in Table 3, the results indicate that (1) the mean depression scores of different personality temperament types were ranked in the following order: melancholic temperament (M = 2.12) > bilious temperament (M = 1.21) > phlegmatic temperament (M = 1.11) > sanguine temperament (M = 0.73); (2) the mean depression scores of different survival situations were ranked as follows: adversity (M = 1.56) > dilemma (M = 1.15) > prosperity (M = 0.89). Additionally, a Chi-Square test was conducted using SPSS—Analyze–Descriptive Statistics–Crosstabs to analyze depression rates under different survival situations and personality temperament types. As presented in Table 4, the results show that (1) the Chi-Square test for survival situations was significant (*p* < 0.001), with depression rates ranked as follows: adversity (24.09%) > dilemma (18.22%) > prosperity (14.27%), and (2) the depression rates of different personality temperament types were ranked in the following order: melancholic temperament (36.05%) > bilious temperament (19.52%) > phlegmatic temperament (17.31%) > sanguine temperament (10.54%).

**Table 3:** One-Way ANVOA results to compare the mean scores of depression in terms of survival situations and personality temperament types

	M	SD	*df*	Mean Square	F	Sig
SS						
Prosperity	0.89	1.549	2	95.583	30.062	0.000***
Dilemma	1.15	1.750				
Adversity	1.56	2.225				
PTT						
Bilious temperament	1.21	1.830	3	300.892	99.343	0.000***
Sanguine temperament	0.73	1.396				
Phlegmatic temperament	1.11	1.742				
Melancholic temperament	2.13	2.259				

***p < 0.001 (2-tails), indicating significance at the 0.1% level, the same below

**Table 4:** Chi-square analysis on depression by survival situations and personality temperament types

Variables	Depression		
	N (%)	χ^2^	*p*
SS			
Prosperity	161 (14.27)	27.702	0.000***
Dilemma	527 (18.22)		
Adversity	165 (24.09)		
PTT			
Bilious temperament	129 (19.52)	202.571	0.000***
Sanguine temperament	156 (10.54)		
Phlegmatic temperament	329 (17.31)		
Melancholic temperament	239 (36.05)		

### Binary logistic regression analysis of life stress on depression

To investigate the association between life stress and depression, binary logistic regression analysis was conducted using SPSS-Analyze-regression-binary logistic. The results (presented in Table 5) indicate that two types of life stressors were significantly associated with depression: stimulus frustration (OR = 2.634, *p* < 0.001), including frustration, difficulty in life, academic frustration, and bad communication, and multiple loading (OR = 3.696, *p* < 0.001), including stress inheritance, academic burden, family pressure, and heavy affairs. The significant stress responses on depression were cognitive response (OR = 9.758, *p* < 0.001), including confusion, blank thinking, negative evaluation, and inability to relieve, emotional response (OR = 3.894, *p* < 0.001), including bad mood, depression and sadness, emotional impulse, anxiety, and annoyance, behavioral reaction (OR = 2.990, *p* < 0.001), including behavior loss of control, self-abuse and self-injury, irritation, alcohol and tobacco anesthesia, and physiological reaction (OR = 3.444, *p* < 0.001), indicating physical injury, cold infection, allergy, discomfort. However, other stressors such as life changes and self-imposed stress were not found to be significant (*p* > 0.05). The model achieved an R^2^ of 0.438 (43.80%). The formula obtained from this study to predict the association between life stress and depression is Logit(depression) =  − 3.414 + 0.968 stimulus frustration + 1.307 multiple loading + 1.237 physiological response + 1.359 emotional response + 1.095 behavioral response + 2.278 cognitive response.

**Table 5:** Binary logistic regression estimates for life stress and depression

	B	SE	Wald	*df*	Sig	Exp(B)
Constant	− 3.414	0.090	1447.670	1	0.000***	0.033
Stimulus frustration	0.968	0.219	19.464	1	0.000***	2.634
Multiple loading	1.307	0.236	30.615	1	0.000***	3.696
Physiological response	1.237	0.260	22.678	1	0.000***	3.444
Emotional response	1.359	0.192	50.068	1	0.000***	3.894
Behavioral response	1.095	0.280	15.279	1	0.000***	2.990
Cognitive response	2.278	0.202	127.396	1	0.000***	9.758

### The moderating interactive effects of personality temperament and survival situation on depression caused by life stress

**Fig. 2:**
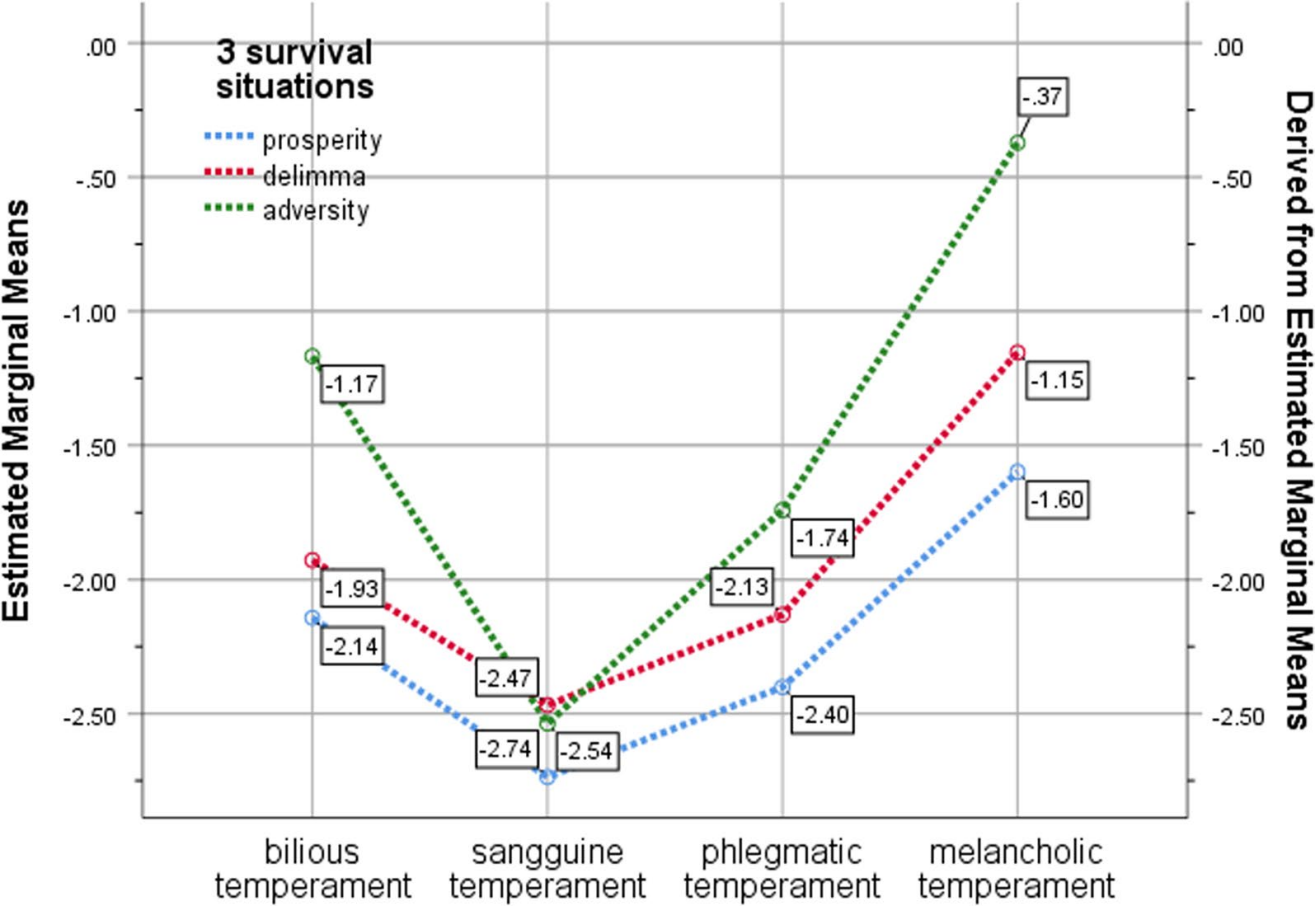
Estimated marginal means of depression under survival situations and personality temperament types

The present study investigated the moderating interactive effects of survival situations and personality temperament types on depression caused by life stress. A General Linear Model was used to conduct Univariate analysis with Logit(depression) as the dependent variable, survival situations and personality temperament types as fixed factors, and survival situations*temperament types as the interactive variable. The results (see Table 6, Fig. 2) indicat that both survival situations and personality temperament types had significant moderating effects (*p* < 0.000) on depression caused by life stress. Additionally, the interactive effects between survival situations and personality temperament types were also significant (*p* < 0.000). Specifically, in prosperity, the depression levels of different personality types ranked in descending order as melancholic temperament (− 1.60) > bilious temperament (− 2.14) > phlegmatic temperament (− 2.40) > sanguine temperament (− 2.74). In dilemma, the depression levels of different personality types ranked in descending order as melancholic temperament (− 1.15) > bilious temperament (− 1.93) > phlegmatic temperament (− 2.13) > sanguine temperament (− 2.47). In adversity, the depression levels of different personality types ranked in descending order as melancholic temperament (− 0.37) > bilious temperament (− 1.17) > phlegmatic temperament (− 1.74) > sanguine temperament (− 2.54).

**Table 6:** Tests of between-subjects effects of survival situations and personality temperament

Source	Type III sum of squares	*df*	mean square	F	Sig
Corrected Model	1215.059	11	110.460	53.123	0.000***
Intercept	9444.370	1	9444.370	4542.024	0.000***
3SS	202.724	2	101.362	48.747	0.000***
4PTT	815.581	3	271.860	130.744	0.000***
3SS * 4PTT	68.924	6	11.487	5.525	0.000***
Error	9758.299	4693	2.079		
Total	31,425.900	4705			
Corrected Total	10,973.358	4704

### Moderated mediation analysis

#### A mediation model

**Fig. 3:**
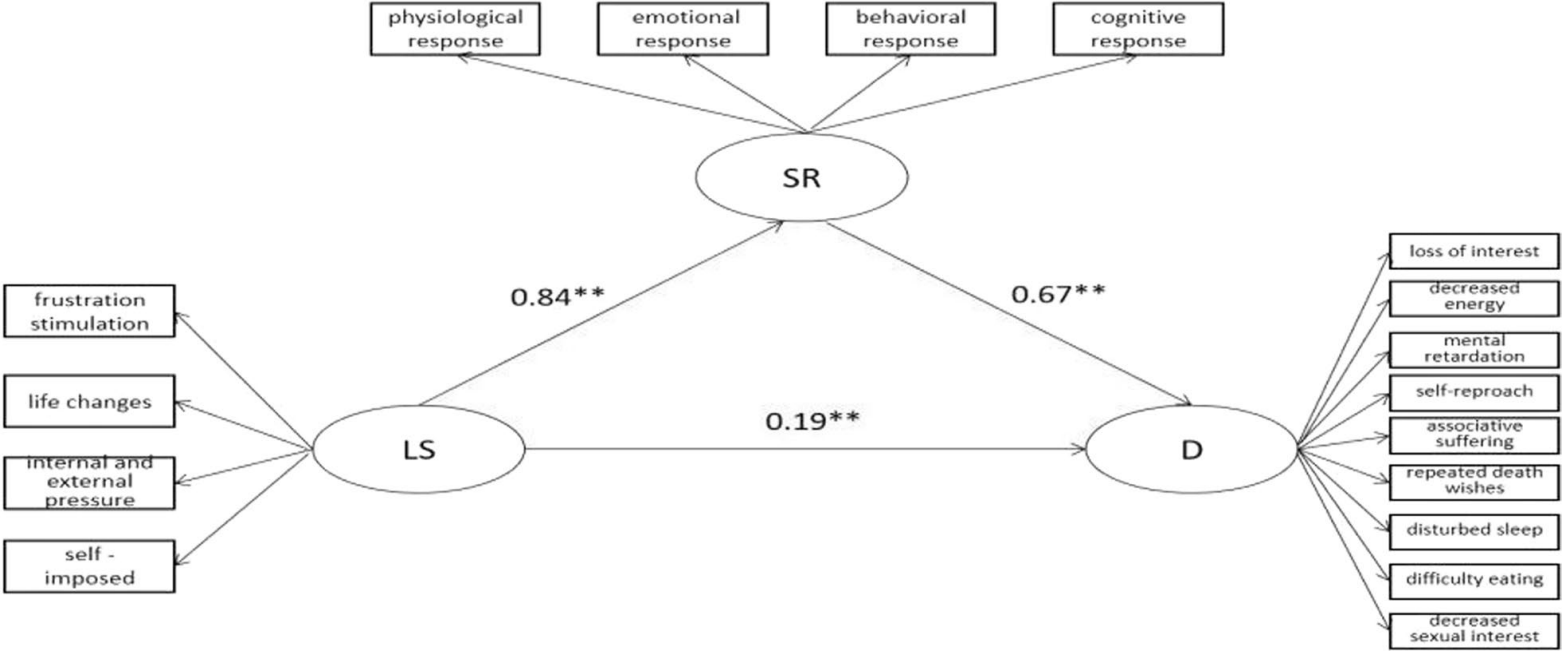
The partial mediation model

We utilized a structural equation model (SEM) with maximum likelihood estimation in AMOS 23.0 to examine a mediation model. Our results indicate that the model fitted well (see Table 7) [22]. Specifically, as shown in Table 8 and Fig. 3, our analyses revealed significant direct (β = 0.19, *p* < 0.001) and indirect (β = 0.56, *p* < 0.001) effects of life stressors on depression.

**Table 7:** Index evaluation system and fitting results of overall structural equation model

Model fit	χ^2^/*DF*	RMSEA	GFI	CFI	TLI	NFI	AGFI
Value	6.795	0.035	0.984	0.971	0.973	0.978	0.974

χ^2^/*DF*, chi-square/degree of freedom, its relatively acceptable value is less than 8.000; RMSEA, the root mean square error of approximation, its acceptable value is less than 0.050; GFI, goodness-of-fit index; CFI, comparative fit index; TLI, Tucker–Lewis index; NFI, normed fit index; AGFI, adjusted goodness-of-fit index, their acceptable value is greater than 0.900

**Table 8:** Total, direct and indirect effects of life stressors on depression

Effect	Path	Estimated β	95% CI	
			Lower	Upper
Total effect	LS–D	0.75	0.715	0.789
Direct effect	LS–D	0.19	0.085	0.293
Indirect effect	LS–SR–D	0.56	0.475	0.653

#### A moderated mediation model

**Fig. 4:**
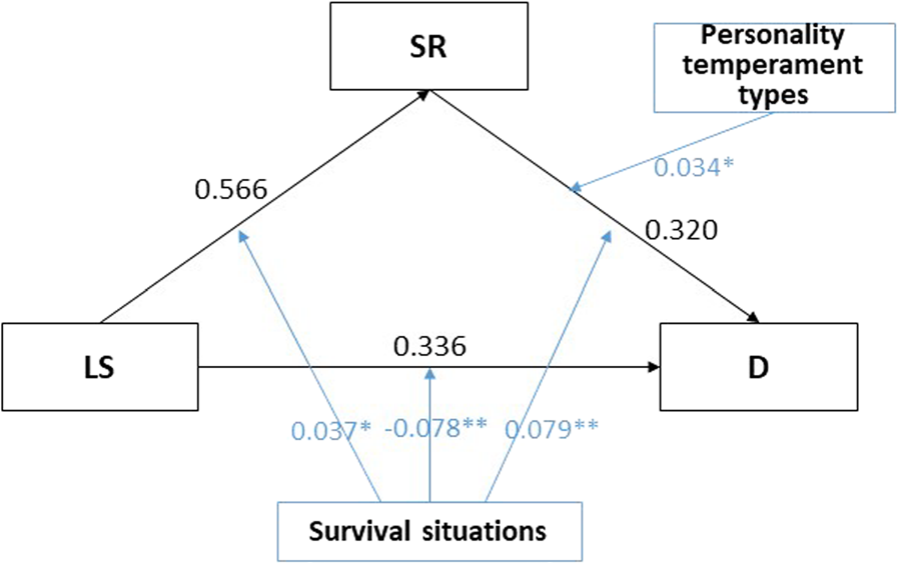
Moderated mediation model

We utilized AMOS multiple group analysis to investigate the moderating effects of survival situations and personality temperament types in a moderated mediation model. Our analyses revealed a significant CHIDIST value (*p* < 0.001), indicating the presence of a moderating effect (see Table 9). To confirm the moderated paths, we used Model 76 of the Hayes SPSS Process [27]. The results (see Table 10, Fig. 4) indicate that survival situations moderated three paths: LS–SR (b = 0.037, *p* < 0.05, 95% CI [0.007, 0.067]), SR–D (b = 0.079, *p* < 0.01, 95% CI [0.031, 0.127]), LS–D (b =  − 0.078, *p* < 0.01, 95% CI [− 0.124, − 0.032]), while personality temperament types moderated one path SR–D (b = 0.034, *p* < 0.05, 95% CI [0.005, 0.063]).

**Table 9:** Multiple group analysis for survival situations and personality temperament types

STRUCKTURAL WEIGHTS	CMIN	DF	CHIDIST
Survival situations multiple analysis	1966.273	427	0.000***
Personality temperament types multiple analysis	1077.402	316	0.000***

**Table 10:** Moderating effects of survival situations and personality temperament

Outcome	Predictor	b	SE	*p*	95% CI	
					Lower	Upper
SR	LS*SS	0.037	0.015	0.016*	0.007	0.067
	LS*PTT	0.008	0.010	0.428	− 0.013	0.030
D	LS*SS	− 0.078	0.024	0.001**	− 0.124	− 0.032
	SR*SS	0.079	0.025	0.001**	0.031	0.127
	LS*PTT	0.009	0.015	0.568	− 0.021	0.038
	SR*PTT	0.034	0.015	0.023*	0.005	0.063

*p < 0.05 (2-tails), indicating significance at the 5% level

## Discussion

The purpose of this study was to explore the effects of survival situation and personality temperament on depression caused by life stress. Data were collected through the self-assessment questionnaire survey and analyzed by SPSSv26, PROCESSv3.3 and AMOSv23. The study confirmed that life stress is an important cause of depression. There were significantly correlated relationship among life stressors, stress response, survival situation, personality temperament and depression. Survival situations and personality temperament types played significant moderating roles on depression caused by life stress.

Firstly, the results of the study confirmed the first hypothesis. The depression rate of vocational college students was high (18.10%), which was consistent with some researches [28, 29] and also consistent with practical experience. According to the Blue Book on Depression of China in 2022, the prevalence rate of depression among adolescents was 15–20% [30]. Long-term depression makes students in a state of impaired social function, affects their life and academic performance [31], damages their physical and mental health, impedes their healthy growth.

Secondly, the results of the study confirmed the second hypothesis. The degrees and rates of depression in dilemma and adversity were higher than those in prosperity, which was consistent with the existing research results. For example, under the background of the COVID-19 epidemic, students' psychological pressure increased, leading to the occurrence of depression [32]. Dilemma and adversity can create chronic stress, which will worsen the depression. The depression levels and rates of melancholic and bilious students are higher than those of phlegmatic and sanguine students. The possible reason for the occurrence of such a result is that the neurotic level of melancholic and bilious temperament is relatively high, one with high neuroticism are prone to be depressive [33]. From general perspective, the proportion of students in dilemma and adversity was 85.44%, It can be concluded that the survival situation of most vocational college students are dire. Recently, the Country issued Opinions on Deepening the Reform of the Construction of Modern Vocational Education System [34] which will further eliminate social prejudice against higher vocational colleges and students and provide them with positive social support from a macro level [35].

Thirdly, The results of the study confirmed the third hypothesis. Partial mediation model showed that on one hand, life stressors can directly induce depressive episodes, stimulation frustration and multiple loading were the main stressors for vocational students. Long-term frustration in studies, improper management of interpersonal relationship and increasing employment pressure cause heavy psychological burden on students, who can not find a sense of belonging, with a result of depression [36]. On the other hand, life stressors can induce depressive episodes through stress response [37], which further verifies the diathesis-stress theory of depression. Stressors stimulates depressive susceptibility qualities, such as false attribution, sensitive constitution and impulsive personality, resulting in depression [8].

Moderated mediation model showed that survival situations moderate three paths. This is consistent with our forecast. Survival situation is the background and environment of individual growth and development and has a permeable influence. It is an important external regulatory factor affecting the growth and development of students. The moderating effect of personality temperament occurs in the path of "stress response-depression", which is consistent with the consistency theory: when personality characteristics are matched with stress content, depression is prone to occur [4]. Personality temperament is an important internal moderating factor. Survival situation and personality temperament play an interactive role in the "stress response-depression" path, which is consistent with the results of some studies [38, 39] and further confirms the interactive model of diathesis-stress theory.

### Limitations and improvement

This study has several limitations. Firstly, the convenient sampling method was used to select only one higher vocational college, and the gender ratio of the sample varied greatly, which may affect the representativeness of the findings. Therefore, a random sampling method and multiple higher vocational colleges should be used in future research. Secondly, depression is influenced by many internal and external factors, but only two of them were selected for this study, which limits the comprehensiveness of the results. Thus, more factors such as psychological capital and social support will be included to enhance the comprehensiveness of the study. Thirdly, self-report questionnaires were used to collect data, which may affect the accuracy of the findings. It is necessary to collect data from both students themselves and those around them to improve the accuracy of the findings. Fourthly, this study used a cross-sectional design, which cannot establish causality. A longitudinal study should be used to explore causality.

### Strengths

Despite the aforementioned limitations, this study expanded its research scope by taking higher vocational students as the research object. The survival situation was classified into three types, which addressed a research gap. Moreover, the study constructed a moderated mediation model that has significant implications for mental health education in higher vocational colleges.

## Conclusion

This study takes higher vocational college students as the research object and divide survival situation into three categories, addressing the research gap. It has confirmed the mechanism of survival situation and personality temperament in the process of depression caused by life stress. Survival situation moderates three paths of the mediation model, personality temperament moderates one path of the mediation, they play an interactive role in the "stress response-depression" path. Melancholic temperament under dilemma and adversity is a risk factor for depressive episode caused by life stress [40], sanguine temperament under prosperity is a protective factor for depressive episode caused by life stress. The results indict that higher vocational colleges should create a positive growth environment and social support, guide students to overcome temperament disadvantages and enhance students’ emotional regulation ability to relieve students' depression, promoting students' healthy growth.

## Data Availability

To protect the privacy of students and ensure the confidentiality of the data, the datasets used and analyzed during the study are not publicly available but can be gained from the corresponding author on reasonable request.
